# Supplementary material for: Spermidine Enhances Mitochondrial Bioenergetics in Young and Aged Human-Induced Pluripotent Stem Cell-Derived Neurons
Source: Antioxidants (Basel). 2024 Dec 4;13(12):1482. doi: 10.3390/antiox13121482 (PMC11673406; doi:10.3390/antiox13121482)
Supplement: Supplementary file 1 [file antioxidants-13-01482-s001.zip › antioxidants-3262400-supplementary.pdf]

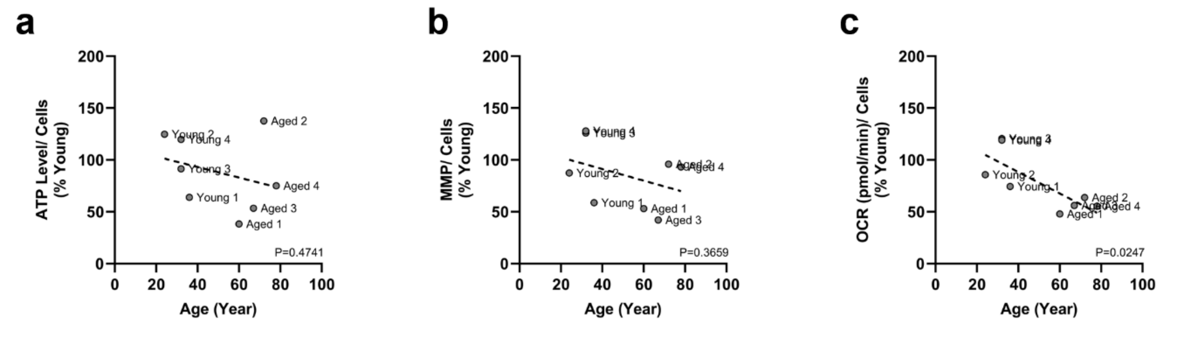


**Supplementary Figure S1.** Correlations between age and bioenergetic parameters in young and aged iPSC-derived neurons. Linear regression analyses were performed between **(a)** ATP level versus age, **(b)** MMP versus age, and **(c)** basal OCR versus age. Values are shown as the mean for each donor in the percentage of all donors. The p-values from simple linear regression tests are indicated on each graph. ATP: adenosine triphosphate; MMP: mitochondrial membrane potential, OCR: oxygen consumption rate.

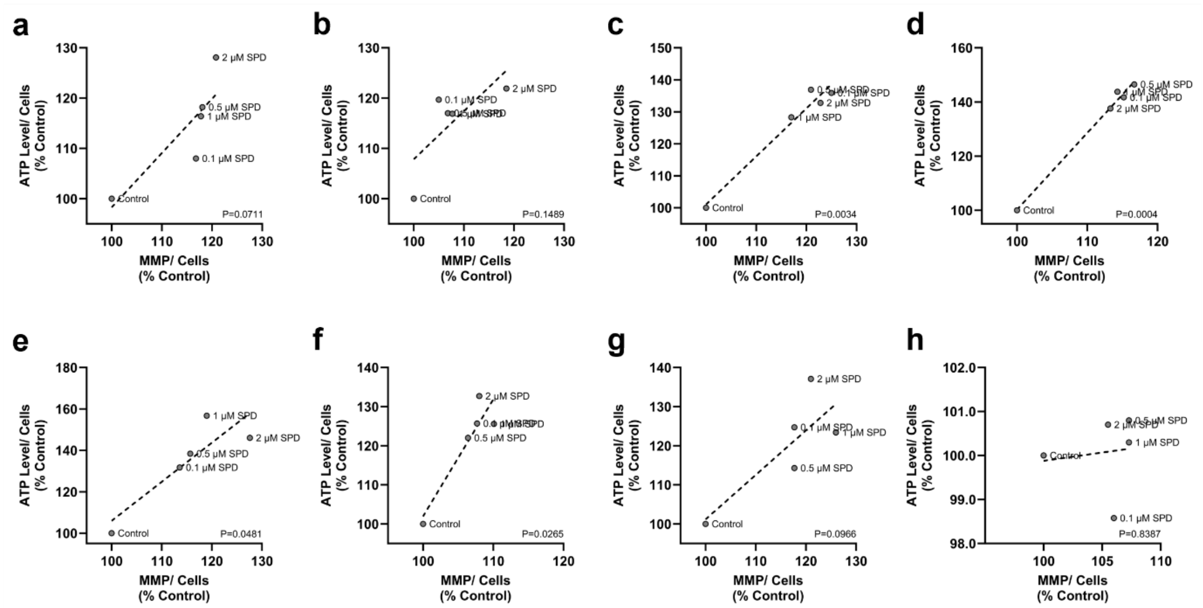


**Supplementary Figure S2.** Correlations between bioenergetic parameters and SPD treatment in young and aged iPSC-derived neurons. Linear regression analyses were conducted to assess the relationship between ATP levels and MMP across various SPD concentrations (0.1 μM, 0.5 μM, 1 μM, 2 μM) in neurons from **(a)** young donor 1, **(b)** young donor 2, **(c)** young donor 3, **(d)** young donor 4, **(e)** aged donor 1, **(f)** aged donor 2, **(g)** aged donor 3, and **(h)** aged donor 4. Values are shown as the mean percentage of the control (untreated control condition). The p-values from simple linear regression tests are indicated on each graph. ATP: adenosine triphosphate; MMP: mitochondrial membrane potential, SPD: spermidine.
